# Dissecting the PI3K Signaling Axis in Pediatric Solid Tumors: Novel Targets for Clinical Integration

**DOI:** 10.3389/fonc.2013.00093

**Published:** 2013-04-23

**Authors:** Amos H. P. Loh, Rachel C. Brennan, Walter H. Lang, Robert J. Hickey, Linda H. Malkas, John A. Sandoval

**Affiliations:** ^1^Department of Surgery, St. Jude Children’s Research HospitalMemphis, TN, USA; ^2^Department of Oncology, St. Jude Children’s Research HospitalMemphis, TN, USA; ^3^City of Hope Comprehensive Cancer CenterDuarte, CA, USA

**Keywords:** PI3K, Akt, mTOR, pediatric solid tumors

## Abstract

Children with solid tumors represent a unique population. Recent improvements in pediatric solid tumor survival rates have been confined to low- and moderate-risk cancers, whereas minimal to no notable improvement in survival have been observed in high-risk and advanced-stage childhood tumors. Treatments for patients with advanced disease are rarely curative, and responses to therapy are often followed by relapse, which highlights the large unmet need for novel therapies. Recent advances in cancer treatment have focused on personalized therapy, whereby patients are treated with agents that best target the molecular drivers of their disease. Thus, a better understanding of the pathways that drive cancer or drug resistance is of critical importance. One such example is the phosphatidylinositol 3-kinase (PI3K)/Akt/mammalian target of rapamycin (mTOR) pathway, which is activated in many solid cancer patients and represents a target for therapy. PI3K/Akt/mTOR pathway activation has also been observed in tumors resistant to agents targeting upstream receptor tyrosine kinases (RTKs). Agents that target this pathway have the potential to shut down survival pathways, and are being explored both in the setting of pathway-activating mutations and for their ability to restore sensitivity to upstream signaling targeted agents. Here, we examine the role of the PI3K/Akt/mTOR pathway in pediatric solid tumors, review the novel agents being explored to target this pathway, and explore the potential role of the inhibition of this pathway in the clinical development of these agents in children.

## Introduction

### The PI3K signaling pathway

Phosphatidylinositol 3-kinases (PI3Ks) are lipid kinases that phosphorylate the inositol ring of phosphoinositides. They generate second messengers that control cell proliferation, survival, motility, and morphological changes. They are grouped into three classes according to structure, substrate specificity, and tissue distribution. Class I PI3Ks are activated by cell surface receptors – Class IA by receptor tyrosine kinases (RTKs) and Class IB by G-protein-coupled receptors. They are involved in cell growth, proliferation, and survival. Class II PI3Ks are activated by RTKs, cytokine receptors, and integrins, and may have a role in regulation of membrane trafficking and receptor internalization. Class III PI3Ks utilize only phosphatidylinositol as substrate. They regulate downstream targets in response to amino-acid availability and cellular stress, and are involved in cell survival and growth.

Class IA PI3Ks are the most commonly implicated in cell division and tumorigenesis. They are composed of heterodimers of a p110α catalytic subunit, and a p85 α regulatory subunit that maintains the former in a quiescent state. The SH2 domain (src oncogene homology-2 domain) of the p85α subunit binds to growth factor receptor and non-receptor protein tyrosine kinases. These include epidermal growth factor receptor (EGFR), platelet-derived growth factor receptor (PDGFR), fibroblast growth factor receptor (FGFR), vascular endothelial growth factor receptor (VEGFR), insulin-like growth factor 1 receptor (IGF-1R), interleukin receptors, interferon receptors, and integrin receptors. This binding releases the p110α subunit inhibition and recruits the complex from the cytoplasm to receptor sites on the inner cell membrane (Jiang and Liu, 2008).

**Figure 1:**
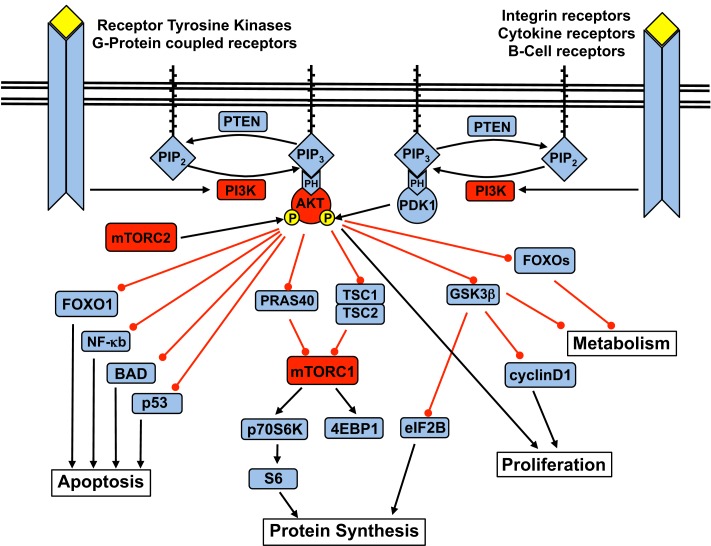
**The PI3K signaling pathway and the downstream targets of Akt**.

Activation of the p110α subunit leads to phosphorylation of its substrate phosphatidylinositol 4,5-bisphosphate (PIP2) at the 3-position of the inositol ring, forming the second messenger phosphatidylinositol 3,4,5-triphosphate (PIP3). PIP3 – which is degraded by PTEN, as well as SHIP1 and SHIP2 – recruits Pleckstrin Homology (PH) domain-containing proteins to cellular membranes. Among them is Akt, also known as protein kinase B (PKB). At the membrane, PKB/Akt is phosphorylated at Thr308 by PDK1 and at Ser473 by the mammalian target of Rapamycin (mTOR)C2 complex. Upon activation, Akt moves to the cytoplasm and nucleus and phosphorylates multiple downstream targets involved in the regulation of various cellular functions (Figure 1).

Key downstream cellular processes induced by Akt include increased cell metabolism and glycogen synthesis via inhibition of the FOXO (forkhead) family and glycogen synthase kinase 3 (GSK3); blockade of apoptosis via inhibition of p53, NF-κB, and BAD (Bcl2-antagonist of cell death); and increased protein synthesis and cell proliferation via activation of the mTOR complex 1 (mTOR-raptor kinase complex, or mTORC1) (Carnero, 2010).

### PI3K pathway mutations in human cancer

Constitutive activation due to deregulation of the PI3K pathway has been implicated in multiple human cancers (Hennessy et al., 2005). The commonest are activating point mutations of PI3K, inactivation of the tumor suppressor PTEN, and amplification of the Akt gene.

Gain of function mutations of PI3K have been observed in many human adult cancers, including ovarian, breast, gastric, and hepatocellular carcinoma. These include increased copy numbers and activating somatic mutations of PI3KCA, the gene encoding the p110α catalytic subunit of PI3K (Samuels and Ericson, 2006; Ligresti et al., 2009). This mutation is more commonly encountered in adult cancers and associated with a more aggressive clinical course, but is less commonly seen in pediatric malignancies. Other mechanisms may be responsible for activating the PI3K pathway in childhood cancers. Other known genetic aberrations involving PI3K include deletion and somatic mutations of PIK3R1, the gene encoding the p85 regulatory subunit, have been noted in colon and ovarian carcinomas, and glioblastoma.

Phosphatase and tensin homolog deleted on chromosome 10 (PTEN) is the antagonist of PI3K. Inactivating mutations of PTEN are encountered in lung, kidney, endometrial and breast carcinomas, melanomas, and glioblastomas. Decreased PTEN expression is negatively correlated with survival outcomes of ovarian, prostate and cervical cancers. Germline mutations of PTEN are associated with the PTEN hamartoma-tumor syndromes (PHTS), a family of hamartomatous polyposis syndromes including Cowden syndrome, Bannayan–Riley–Ruvalcaba syndrome, Proteus syndrome, and Proteus-like syndrome (Chow and Baker, 2006).

Akt gene amplification has been observed in cases of gastric carcinoma, glioblastoma, and gliosarcoma. Amplification of its isoforms have also been reported in various human cancers: Akt2 in pancreatic, ovarian, breast, and head and neck squamous cell carcinomas, and Akt3 in breast and prostate cancers. An activating somatic mutation of Akt1 (Akt-E17K) has been reported in human breast, colorectal, and ovarian cancers. This mutation induces plasma membrane localization, downstream signaling, and transforms cells, and induces leukemia in mice.

As a collective group, brain tumors are the commonest solid tumors in children. The commonest extracranial solid tumors are neuroblastoma (NB), osteosarcoma, Ewing sarcoma family of tumors (ESFT), rhabdomyosarcoma (RMS), germ cell tumors, and Wilms tumor. The overall survival rate of the former four tumors in the advanced stage remains less than 30% despite intensive efforts over the past three decades using multiple therapeutic modalities including chemotherapy, surgery, radiation, autologous bone marrow transplant, and biological agents. The PI3K signaling pathway has been implicated in the tumorigenesis of NB, ESFT, RMS, and osteosarcoma, and this review will focus on these tumor types, and discuss chemotherapeutic agents in current pediatric preclinical and clinical trials.

## PI3K Signaling in Pediatric Solid Tumors

### PI3K signaling in neuroblastoma

Despite current multi-modal treatments comprising chemotherapy, surgical gross total resection, radiotherapy, stem cell transplantation, and immunotherapy, high-risk cases of NB still have a very poor prognosis despite aggressive therapy, often recurring with chemoresistant metastases. This tumor alone accounts for more than 10% of all pediatric cancer deaths. Clearly, new treatment strategies are needed particularly for high-risk patients. This tumor most commonly arises in the retroperitoneum from the adrenal glands and paravertebral sympathetic plexuses. It is highly vascular in nature, and wraps around major abdominal vessels, developing extensive tumor neovasculature. This reflects activation of angiogenic and tumorigenic pathways, many of which are activated through RTKs which trigger PI3K signaling.

Activation of the PI3K signaling pathway has been demonstrated as a common event in NB cells. In an analysis of 39 samples of high-risk tumors, high levels of PI3Kp85 and PI3Kp110 expression were seen in the cytoplasm in 54% of cases (Iżycka-Swieszewska et al., 2010). However, activating gene mutations of PI3KCA and the loss of function mutations of tumor suppressor PTEN are very rarely present in NB, even though they are commonly seen in other human cancers. Sequencing data from 42 primary human NB tumors and 27 cell lines found activating mutations in the PI3KCA gene in only 2.9% of samples, and this mutation rate did not differ significantly between those with and without MYCN amplification (Dam et al., 2006). Using RT-PCR, Muñoz et al. (2004) found homozygous deletions of PTEN in only 2 of 41 (5%) primary tumors, and in none of 12 cell lines.

Phosphatidylinositol 3-kinase pathway activation in NB may take place via different mechanisms. Decreased PTEN expression may play a role in inducing PI3K signaling in NB tumorigenesis via decreased negative regulation on the p110 subunit. This hypothesis is supported by observations of decreased PTEN protein expression seen on immunohistochemical analysis. A decrease in the ratio of PTEN to phospho-Akt protein expression was observed in undifferentiated tumors (Qiao et al., 2005). Also, decreased intensity of nuclear expression of PTEN has been observed in up to a third of cases (Iżycka-Swieszewska et al., 2010).

Correspondingly, activation of downstream targets of the PI3K pathway have been demonstrated in NB tumor cells. Akt activation has been shown to correlate with a poorer prognostic outcome in NB, and is associated with other markers of aggressive disease, including NMYC amplification, advanced disease stage and unfavorable histology (Opel et al., 2007). On immunohistochemistry, intense phospho-Akt staining is observed in undifferentiated and poorly differentiated neuroblasts. The degree of neuroblast differentiation has been observed to correlate inversely with the expression of phospho-Akt – which is seen almost exclusively in the cytoplasm. Akt phosphorylation activates downstream mTOR kinase. In the same study, a statistically significant association was demonstrated between phospho-Akt and phospho-mTOR expression (Qiao et al., 2005). In a series of 30 primary NB tumor samples, phospho-Akt and mTOR was detected in all samples, but was not seen in the Schwannian stroma and non-malignant adrenal medulla (Johnsen et al., 2008). Our laboratory is presently investigating the significance of differential phosphorylation levels of Akt and mTOR in NB cell lines and xenografts, as well as differential phosphorylation at the key activation sites Thr308 and Ser473.

mTOR is a major controller of cell growth and a negative regulator of autophagy, and its activity in NB cells has been shown to affect cell differentiation by regulating the balance between macromolecule synthesis and degradation. In the first morphoproteomic study performed on three samples of high-risk primary human NB tumors, Brown et al. (2007) found activation of the mTOR/p70S6K, NFkB, and ERK 1/2 pathways. Conversely, decreased activation of the mTOR pathway is associated with increased neuroblastic differentiation. Using mouse NB N2a cells in culture, Zeng et al. showed that downregulation of Akt-mTOR signaling accompanied differentiation of murine NB cells induced by treatment with retinoic acid. This occurred in the same time-dependent pattern as the induction of autophagy as indicated by the relative expression of the autophagy protein LC3 (Zeng and Zhou, 2008).

### PI3K signaling in Ewing sarcoma family of tumors

The ESFTs is thought to arise from primordial bone marrow-derived mesenchymal stem cells, and encompass primitive neuroectodermal tumors, Askin tumor (Ewing sarcoma of chest wall), and extraosseous Ewing sarcomas. They are characterized by distinctive karyotypic abnormalities involving the EWSR1 locus on chromosome 22 band q12, which forms fusion products with different members of the Ets family of transcription factors (de Alava and Gerald, 2000). The commonest translocation t(11;22)(q24;q12), involving Fli-1, is reported in more than 85% of ESFTs. Other common translocations include t(21;22), t(7;22), and t(17;22), involving the ERG, ETV1, and E1AF genes, respectively.

These fusion proteins act as abnormal transcription factors, and also influence post-transcriptional RNA processing. The EWS/Fli-1 fusion product has been shown to transform murine fibroblasts *in vitro* through a mechanism involving IGF-1. In these fibroblasts transformed with the ESW/Fli-1 fusion protein, IGF-1 acts via IRS-1 (Insulin receptor substrate 1), which moves from a low to high phosphorylation state and transmits signals to activate PI3K (Toretsky et al., 1997). Separately, IGF-1 can also activate Ras, which then activates PI3K. IGF-1 is a major survival factor that induces neoplastic transformation and diminished apoptosis, and is implicated in the carcinogenesis of prostate, breast, and other cancers. All ESFTs express the IGF-1 receptor, and IGF-1 is stored in bone matrix, which is found at the site of origin of osseous ESFTs. As such, it is suggested that ESFT tumorigenesis may be induced by paracrine and autocrine stimulation by IGF-1 (Benini et al., 2004), which then activates Ras and PI3K downstream.

The PI3K/Akt pathway mediates survival signals in ESFT via mechanisms involving IGF-1. Downstream activation of PI3K by IGF-1 has been shown to partly mediate inhibition of apoptosis (Kulik et al., 1997; Párrizas et al., 1997), and correspondingly inhibition of PI3K dramatically reduces cell proliferation in Ewing sarcoma cell lines. PI3K inhibition sensitizes these cells to apoptosis by FGF-2 (fibroblast growth factor 2) – a growth and differentiation factor that induces growth arrest in ESFT cells (Hotfilder et al., 2005). Constitutive activation of Akt in a transfected ESFT cell line rendered the cells more resistant to apoptosis by Doxorubicin, implicating the IGF-IR/PI3K/Akt pathway in the survival signaling mechanisms in ESFT cells (Kulik et al., 1997; Toretsky et al., 1999).

The EWS/Fli-1 fusion product also plays a role in regulation of telomerase activity and phospholipase D expression. The latter occurs by activation of cell proliferation via the MEK (mitogen-activated protein kinase)/ERK (extracellular signal-regulated kinase) pathway and the PI3K/Akt pathway (Banno et al., 2001). Constitutive activation of the PI3K and MEK/MAPK pathway has been observed in ESFT cell lines maintained in standard medium (Benini et al., 2004).

Phosphatidylinositol 3-kinase activation has been found to be essential for cellular polarization and elongation, which are the first steps in cell motility, the corresponding initial step in the process of cell invasion and metastasis. In ESFT cells, PI3K/Rac1 activation by bFGF (basic fibroblast growth factor) induced these morphological alterations and also increased cell motility (Kamura et al., 2010). Akt/mTOR signaling is not implicated in this pathway, but is activated instead by IGF-1 (type I insulin-like growth factor) to stimulate F-actin reorganization and induce cell motility in both ESFT and RMS cells (Liu et al., 2008).

Activation of the PI3K pathway is implicated in the development of chemotherapy resistance and is frequently observed when conventional anticancer drugs are used (Yu et al., 2008). PI3K inhibition may serve to decrease chemotherapy resistance, in addition to its direct anticancer effects. PI3K/mTOR inhibitors Wortmannin and LY294002 sensitized ESFT cells in culture to increased apoptosis by various common chemotherapeutic agents. Doxorubicin-induced apoptosis was enhanced when PI3K was inhibited in TC-71 and TC-32 ESFT cells, as evidenced by increased DNA fragmentation, increased caspase-3 activity, and on TUNEL assay (Toretsky et al., 1999; Benini et al., 2004). Treatment of TC-135 Ewing sarcoma cells with Actinomycin D induced significant activation of the MEK/ERK and PI3K/Akt pathways. Inhibition of pAkt with LY294002 enhanced Actinomycin D-induced caspase-dependent cell apoptosis as determined by PARP cleavage assays. This was not seen with p-ERK inhibition. These experiments suggested that the PI3K/Akt pathway was chiefly implicated in the mechanisms of chemotherapeutic resistance to Actinomycin D in ESFT cells (Yamamoto et al., 2009).

### PI3K signaling in rhabdomyosarcoma

Rhabdomyosarcoma has several distinct histological subtypes. Embryonal tumors are the most frequent, and account for 60–70% of all childhood RMS. They arise most frequently in the head and neck, and the region of the genitourinary tract. Alveolar tumors are less common but behave more aggressively, and are more common in the extremity, trunk, and perineal regions. Anaplastic tumors are rarely seen in children. Distinctive molecular abnormalities characterize the two commonest types of RMS. Up to 80% of alveolar tumors possess the t(2;13)(q35;q14) or t(1;13)(p36;q14) chromosomal translocations, involving the FOXO1 (FKHR) gene on chromosome 13 and the PAX 3 or PAX 7 genes, respectively (Shapiro et al., 1993). These fusion proteins function as transcription factors that play a role in cell cycle dysregulation, and play a major role in the tumorigenesis of RMS.

Phosphatidylinositol 3-kinase/Akt signaling has been known to play a direct role in regulating myogenesis and myoblast differentiation (Kaliman et al., 1996; Jiang et al., 1999; Li et al., 2000). In normal physiological regulation, PI3K/Akt activation enhances cell proliferation and migration via antagonism of the winged-helix family of forkhead transcription factors. PI3K activation induces phosphorylation, nuclear export, and transcriptional inactivation of FOXO1, FOXO3, FOXO4, and FOXO6. Akt directly phosphorylates FOXO1 at Thr24 and Ser253, negatively regulating its transcription by promoting export from the nucleus. This decreases expression of downstream target genes, which include the pro-apoptotic Fas ligand, TRAIL, and Bim (Biggs et al., 1999; Brunet et al., 1999). FOXO expression represses the transcription of D-type cyclins, which regulate cell cycle G1 progression and function as key sensors for mitogenic growth factors (Zhang et al., 2004). Thus, the net effect of physiological PI3K/Akt activation is enhancement of cell proliferation and migration.

In RMS, cellular proliferation occurs via distinct – though not necessarily independent – mechanisms involving its characteristic fusion products. In cells possessing the PAX7-FOXO1 fusion transcript, phospho-Akt is upregulated with increased nuclear localization. This upregulates NF-kappaB, which in turn suppresses MyoD1-mediated terminal myogenic differentiation via cyclin-dependent kinase 4 (cdk4). In a panel of 21 human alveolar RMS samples, 85% of the tumors displayed nuclear or cytoplasmic expression of cdk4. It is suggested that through this mechanism, inappropriately sustained expression of PAX7-FOXO1 fusion transcripts maintains skeletal muscle precursors in an undifferentiated state (Charytonowicz et al., 2012). PAX3-FOXO1 activates PI3K/Akt signaling by downregulating PTEN. In RMS cell lines, PAX3-FOXO1 inhibition induced PTEN upregulation. Conversely in C2C12 myoblasts, PAX3 induction downregulated PTEN (Li et al., 2007). In addition, physiological regulators of muscle differentiation are deranged in RMS. Expression of FOXO1 is significantly higher in RMS, even when compared with other tumors like ESFT. MyoD, which is required for proper skeletal muscle differentiation, is widely expressed in RMS cells, but functionally deficient (Tapscott et al., 1993).

Insulin-like growth factor mediates its effects on muscle differentiation via the PI3K/Akt pathway by upregulating endogenous myogenin expression. RMS is characterized by high levels of IGF-2, which is produced in an autocrine manner (Minniti et al., 1995). This IGF-2 overexpression drives Akt phosphorylation. IGF-2 induction of Akt is regulated by differential phosphorylation at Ser473, rather than at Thr308 where high levels of Akt phosphorylation are seen in all RMS cell lines. This IGF-2-overexpression-mediated Akt phosphorylation at Ser473 is modulated by PTEN protein expression levels, but the exact mechanism by which PTEN regulates Ser473 phosphorylation of Akt is unclear. In an autocrine loop, the PAX3-FOXO1 fusion transcript transactivates the IGF-1R promoter, further potentiating the effects of IGF on PI3K/Akt activation. In sarcoma-derived cell lines, the PAX3-FOXO1 fusion protein induces a more significant rise in IGF-1 levels as compared with PAX3 alone (Ayalon et al., 2001).

Phosphorylation levels of many receptor and non-RTKs and serine/threonine kinases in the mTOR and PKC pathways are elevated in RMS as compared with normal muscle tissue. These are strongly implicated in the development and tumor progression of RMS. In an analysis using 64-core primary tumor tissue microarrays from the Children’s Oncology Group (COG), mTOR phosphorylation levels were elevated in 60% of alveolar and 68% of embryonal cases; PKC isozymes were upregulated in up to 69% of alveolar and 72% of embryonal cases. However, in the study, these were not significantly correlated with clinicopathologic features (Cen et al., 2007).

### PI3K signaling in osteosarcoma

Osteosarcomas have numerous chromosomal aberrations and complex karyotypes, with chromosomal abnormalities involving members of the PI3K/Akt pathway (Lau et al., 2004; Man et al., 2004). In a series of 59 pediatric patients, PTEN deletions were observed in 12 cases (Freeman et al., 2008). In a series of 98 primary adult osteosarcomas, 40 were found to have at least 1 gene mutation, and among these, 3 novel mutations involving PI3KCA were observed. Yet, samples without PI3KCA mutations have been found to have high levels of pAkt and p4EBP1, suggesting that alternate mechanisms exist for the hyperactivation of PI3K in osteosarcoma (Choy et al., 2012). While the tumorigenesis of this tumor is poorly understood, cancer stem-like cells from human and canine osteosarcoma have been isolated (Gibbs et al., 2005), and used to study the molecular pathways involved in progression of this tumor. The PI3K/Akt pathway has been shown to play an important role in cell cycle regulation and apoptosis of osteosarcoma cancer stem-like cells, with dose-dependent decrease in pAkt expression seen following PI3K inhibition with LY204002. This yields G0/G1 cell cycle arrest and increased apoptosis indicated by activation of caspase-9, caspase-3, and PARP, which are involved in the mitochondrial apoptosis pathway (Gong et al., 2012).

Phosphatidylinositol 3-kinase/Akt regulation in osteosarcoma cells has been modulated by administration of bisphosphonates *in vitro*, with resulting impacts on cell survival, and invasion. In murine osteosarcoma cell lines, bisphosphonates inhibited Ras/PI3K/Akt and Ras/MEK/ERK expression, which produced decreased cell migration, invasion, and adhesion to the extracellular matrix, as well as decreased mRNA expression and activity of matrix metalloproteinases (Tsubaki et al., 2012). These zinc-dependent endopeptidases are major components of the basement membranes and are thought to correlate with tumor invasiveness and metastatic potential. In addition, other agents have shown the ability to regulate downstream targets of the PI3K pathway through alternative mechanisms, independent of PI3K activity. Alendronate, an amino-bisphosphonate commonly used in clinical practice, inhibits Akt phosphorylation at Ser473 and Thr308 in osteosarcoma cell lines. This causes apoptosis by inhibiting the PI3K/Akt/NFκB cell survival pathway at the initial step of PI3K activation (Inoue et al., 2005). Zoledronic acid has been shown to augment the effect of mTOR inhibition by Everolimus in mice models. Everolimus inhibition alone induced marked decrease in the rate of cell cycle phases and mitosis, but further significant additive effects were seen with addition of Zoledronic acid, resulting in downregulation of mTOR downstream signaling involving 4EBP1 and decreased Ras isoprenylation and GTP binding. This yielded decreased tumor development and increased bone mass (Moriceau et al., 2010). These alternative activation pathways may be relevant to mechanisms of tumor escape following treatment with PI3K inhibitors, and warrant equal attention in the overall development of targeted therapy involving this pathway.

## Therapeutic Targeting of PI3K Pathway for Pediatric Solid Tumor Treatment

### Background

As with many clinical advances in medicine, rapamycin, derived from *Streptomyces hygroscopicus*, was initially developed as a fungicidal (Sehgal et al., 1975) and immunosuppressive agent used in the setting of organ transplantation (Calne et al., 1989). Though the anti-proliferative activity of rapamycin as an mTOR inhibitor in preclinical models was also observed, the poor aqueous solubility and chemical stability of this agent made it a poor choice for further development in cancer therapy. However, rapamycin derivatives (“rapalogs”) with improved pharmaceutical properties and comparable efficacy sparked further interest in the use of this drug. Further insights regarding the molecular mechanics of PI3K signaling that promote cancer cell growth and survival expanded the potential targets in this pathway, resulting in a myriad of agents targeting PI3K, Akt, mTOR, and even dual PI3K/mTOR inhibition. A recent review highlighted many of these PI3K-Akt pathway inhibitors in clinical development for treating adult cancers (Engelman, 2009). Of note, the Engelman review also concisely addressed the challenges of identifying “sensitive” populations with particular pathway abnormalities that are most likely to benefit from the targeted therapy and then providing the right combination or complementation with targeted therapy to overcome the potential for resistance due to signaling feedback loops within this pathway. A full discussion of the success and/or failure of these trials is beyond the scope of this review. While the clinical route to targeting PI3K signaling in pediatric solid malignancies shares many of the same challenges, we cannot directly extrapolate from adult studies. Pediatric trials focusing on this pathway face additional challenges of accrual and differences in the molecular signature of pediatric tumors, requiring thoughtful and efficient utilization of existing preclinical evidence to prioritize the most active therapies (Table 1).

**Table 1:** **Current pediatric clinical trials targeting the PI3K pathway**.

Drug	Mechanism	Study type	Study phase	Study aims/findings
Perifosine	Akt inhibition	Single agent dose escalation study (NCT00776867)	I	Determine molecular predictors of response (PI3K/AKT/mTOR and RAS/MAPK signaling, cell cycle markers)
Perifosine + Temsirolimus	Akt + mTOR inhibition	Single arm study (NCT01049841)	I	Establish preliminary data on efficacy of combination therapy; determine molecular predictors of response
MK-2206	Akt (allosteric) inhibition	Dose escalation study followed by treatment at MTD (NCT01231919)	I/II	Evaluate biological activity in tumor and PBMC in recurrent or refractory leukemia, lymphoma or solid tumors
Everolimus	mTOR inhibition	Single agent dose escalation study (Fouladi et al., 2007)	I	MTD dose level correlated with degree of mTOR inhibition in PBMC
Deforolimus	mTOR inhibition	Single agent dose escalation study (Hartford et al., 2009)	I	Toxicity profile similar to other mTOR inhibitors; no objective disease responses seen
Ridaforolimus	mTOR inhibition	Single agent safety study (NCT01431534), and in combination with Dalotuzumab (NCT01431547)	I	Establish MTD, and recommended Phase 2 dose and potential efficacy of the combination therapy
Temsirolimus	mTOR inhibition	Single agent dose escalation study (Spunt et al., 2011)	I	Well tolerated; MTD not identified
Temsirolimus	mTOR inhibition	Single agent study (Geoerger et al., 2012)	II	Did not meet criteria to continue study as single agent; significant number with disease stabilization
Temsirolimus	mTOR inhibition	Combination therapy with Cixutumumab (Naing et al., 2011)	I	Well tolerated
Temsirolimus	mTOR inhibition	Combination therapy with liposomal Doxorubicin (Thornton et al., 2013)	I	MTD defined, toxicity profile acceptable; combination therapy increased exposure to the active metabolite of Temsirolimus
Temsirolimus	mTOR inhibition	Combination therapy with Cixutumumab (NCT01614795)	II	Evaluate IGF-1R, insulin receptor, ERK, RON, mTOR activation in refractory or recurrent sarcomas
Temsirolimus	mTOR inhibition	Combination with Irinotecan, Temozolomide (NCT01141244/COG-ADVL0918)	I	In younger patients with recurrent or refractory solid tumors; establish MTD and efficacy of combination therapy
Rapamycin (Sirolimus)	mTOR inhibition	Combination with Topotecan, Cyclophosphamide (NCT01670175)	I	Determine dose-limiting toxicity, antitumor activity, biologic and anti-angiogenic effects of drug combination
Rapamycin (Sirolimus)	mTOR inhibition	Single arm efficacy study (NCT01265030)	I	Determine tolerability of preoperative administration in children and young adults with desmoid-type fibromatosis; evaluate disease recurrence, pain improvement, mTOR pathway activation
Rapamycin (Sirolimus)	mTOR inhibition	Combination with Vinblastine (NCT01135563)	I	In relapsed solid tumors including selected brain tumors and lymphoma; establish MTD and response to combination therapy
Enzastaurin	PKC-β inhibition	Fine et al. (2005)	II	In recurrent glioblastoma multiforme; good preliminary radiographic response
Enzastaurin	PKC-β inhibition	Randomized trial comparing Lomustine (Wick et al., 2010)	III	No significant differences on interim analysis; early study termination

*MTD, maximum-tolerated dose; PBMC, peripheral blood mononuclear cells*.

### PI3K/Akt inhibition in pediatric solid malignancies

Perifosine targets the lipid-binding PH domain of Akt and inhibits the translocation of Akt to the cell membrane, an essential step for Akt activation (Richardson et al., 2012). It decreases Akt phosphorylation and increases caspase-dependent apoptosis in NB cell lines, inhibits growth of NB xenografts, and overcomes RTK/ligand-mediated chemoresistance (Li et al., 2010, 2011). It is currently being studied in two Phase I clinical trials in children with recurrent or refractory solid tumors. In the single agent trial (ClinicalTrials.gov identifier NCT00776867), the maximum-tolerated dose (MTD) has not yet been reached. The study opened in 2008 and is expected to accrue 36 participants through 2013. In the second study (NCT01049841), patients are treated with a combination of perifosine and the mTOR-inhibitor temsirolimus based on preclinical data showing synergy of the two agents. It is hypothesized that direct Akt inhibition may overcome Akt activation secondary to mTOR inhibition in this study population.

A second targeted agent, MK-2206, is a novel allosteric Akt inhibitor that has been tested in adult cancers. In preclinical trials, MK-2206 increased the efficacy of etoposide or rapamycin in NB cell lines (by MTS assay) (Li et al., 2012). Currently the COG is enrolling participants with refractory solid malignancies and recurrent or refractory leukemia (NCT01231919) on a phase I/II trial with MK-2206 to determine the MTD and toxicities of this agent. The study also will evaluate the biological activity of MK-2206 by measuring PI3K/Akt/mTOR signaling in tumor and peripheral blood mononuclear cells (PBMCs) and measure the expression of biomarkers related to Akt activation phenotypes.

There are several PI3K agents currently in adult phase 1/2 clinical trials. Based on favorable preclinical evidence (Maira et al., 2008; Serra et al., 2008; Brachmann et al., 2009; Maira, 2011; Bendell et al., 2012; Koul et al., 2012), pediatric preclinical testing is underway for two of these agents: NVP-BKM-120, a selective inhibitor of PI3K, and NVP-BEZ-235 a dual mTOR and PI3K inhibitor (Federico, 2012). Results obtained in pediatric NB cell lines are promising and testing on xenografts is currently underway (Federico, 2012).

### Targeting mTOR in pediatric solid malignancies

There is significantly more clinical experience with mTOR inhibitors in pediatrics. While there is clear efficacy in preclinical cancer models, the clinical application of these agents remains under investigation.

In 2007, results of a phase I study of everolimus in pediatric patients with refractory solid tumors were published (Fouladi et al., 2007). Continuous oral administration was well tolerated at the MTD of 5 mg/m^2^, a dose level which correlated with significant inhibition of the mTOR pathway signaling in PBMCs. A second mTOR inhibitor, Deforolimus, was subsequently evaluated in a phase I trial in pediatric patients with advanced malignancies (Hartford et al., 2009). The drug was again well tolerated with one partial response and 21 patients with stable disease (12 progressive disease). A similar toxicity and pharmacokinetic profile to other mTOR inhibitors was observed, though no objective disease responses were observed. The third mTOR inhibitor to be evaluated in phase I trials, temsirolimus, followed in 2011 (Spunt et al., 2011). The drug was very well tolerated and an MTD was not identified. After two courses of therapy, 1/13 evaluable patients achieved CR (NB) and 5/13 had stable disease. Based on these results, a phase II trial of temsirolimus in children and adolescents with high-grade glioma (HGG), NB, and RMS was initiated (Geoerger et al., 2012). At a dose of 75 mg/m^2^ administered intravenously once weekly for 12 weeks, no cohort met criteria for continued enrollment with only one partial response reported (NB). However, disease stabilization was observed in 7/17 with HGG, 6/19 with NB, and 1/16 with rhabdomyosarcoma. The most common treatment related adverse effects were thrombocytopenia, hyperlipidemia, and asthenia. While this drug did not meet criteria in this study to continue investigation as a single agent, the promising observation of disease stabilization in heavily pretreated patients with relapsed or refractory disease certainly merits further evaluation in combination therapy. The most recent mTOR inhibitor under evaluation in a phase I clinical trial is Ridaforolimus, both alone (NCT01431534) and in potential combination with Dalotuzumab (NCT01431547).

Several clinical studies are further investigating the impact of mTOR inhibition in combination with other agents in pediatric solid malignancies. Most recently, findings have been published from a Phase I/II study of the use of temsirolimus and liposomal doxorubicin in refractory or recurrent bone or soft tissue sarcomas. Of 15 patients enrolled in the phase I study, 7 were under 21 years of age. Toxicities were manageable, and mostly hematologic. Pharmacokinetics of temsirolimus were not altered by co-administration with liposomal doxorubicin, but increased exposure to its active metabolite, sirolimus. Progression-free survival appeared to be superior to other trials with either agent given alone. The Phase 2 portion of the study is ongoing, along with pharmacodynamics studies to test if temsirolimus overcomes inherent chemoresistance of sarcoma stem cells *in vivo* (Thornton et al., 2013). A Phase I study of the combination of rapamycin (sirolimus), cyclophosphamide, and topotecan, all given by oral administration, in pediatric and young adult patients with relapsed and refractory solid tumors opened in August 2012 (NCT01670175). Sirolimus was paired with vinblastine in a phase I trial for recurrent or refractory solid tumors including central nervous system tumors that opened in 2010 (NCT01135563); no results have been published to date. Another Phase II trial opened in June 2012 to investigate the combination therapy of temsirolimus with Cixutumumab, an IGF-1R inhibitor, in refractory or recurrent sarcomas (NCT01614795, COG-ADVL1221) after promising results were reported in patients within this cohort during the Phase I trial (Naing et al., 2011). While IGF-1R antagonists showed more preclinical promise than clinical efficacy as single agent therapy, they may be useful in combination therapy (Wagner and Maki, 2013). The therapy was well tolerated in the phase I with most common dose-limiting toxicities of mucositis, febrile neutropenia, and thrombocytopenia. Temsirolimus has also been paired with irinotecan and temozolomide (NCT01141244, COG-ADVL0918) in a phase I study for young patients with relapsed or refractory solid tumors. Finally, a pilot study evaluating sirolimus in children and young adults with desmoid-type fibromatosis (NCT01265030) opened in December 2010 and is currently recruiting patients. Rationale for this trial is based on recent evidence that deregulation of the mTOR cell proliferation/survival pathway may play an important role in tumor biology when the APC/B-catenin or WNT/CTNNB1 signaling pathway is disrupted (Laguë et al., 2008; Pressey et al., 2010) as occurs in desmoid tumors.

For patients with hematologic malignancies, there is a phase I trial utilizing temsirolimus in combination with other standard agents for relapsed ALL and NHL (NCT0164197) and another phase I trial investigating the addition of everolimus to multiagent re-induction chemotherapy for pediatric patients with ALL in first bone marrow relapse (NCT01523977). A recent review summarized abnormalities in the PI3K/Akt/mTOR signaling pathway in hematologic malignancies and reviewed the results of preclinical and clinical research supporting the use of these agents for pediatric patients with these cancers (Barrett et al., 2012). Recent preclinical investigation indicates that mTOR inhibition may also disrupt leukemia/stromal interactions, leading to antitumor effect by a different mechanism (Zeng et al., 2012).

In tuberous sclerosis, the associated inactivating mutations of TSC1 and TSC2 result in constitutive activation of the mTOR pathway independent of upstream PI3K/Akt stimulation. For patients with tuberous sclerosis who develop subependymal giant-cell astrocytomas, everolimus therapy has been shown to reduce tumor volume and seizure frequency (Krueger et al., 2010), and with further long-term study, may provide an alternative to surgical resection.

### Challenges associated with individualized PI3K treatment

The development of targeted PI3K therapies will likely lead to the addition of these antagonists to existing therapy options, alone or in combination with background treatments (chemotherapy, surgery, radiotherapy), and eventually offer enhanced treatment schemes to children with solid tumors. Nevertheless, despite the promise emerging from these targeted compounds, the ability to identify patients’ subgroups and response predictive factors will be crucial in obtaining the greatest benefit with minimal toxicity. The utility of biomarkers related to PI3K signaling pathway and the appropriate patient selection based on the expression of relevant therapeutic targets in tumors represents an obstacle regarding the most optimal application of PI3K antitumor agents. For example, PTEN, pAkt, p27, and pS6 were found to be informative markers of the mTOR pathway for patient selection in predicting the response to mTOR inhibitors for renal cell carcinoma (Pantuck et al., 2007). Other molecular patterns evaluating the effects of mTOR inhibitors include overexpression of anti apoptotic proteins (Bcl2) that may serve as a surrogate marker for rapalog resistance (Delbaldo et al., 2011). Lastly, the activation of alternative survival pathways such as deregulated KRAS has been shown to bypass Everolimus-mediated mTOR inhibition (Delbaldo et al., 2011). Given the potential hurdles associated with identifying validated predictive biomarkers for PI3K therapy, it is obvious molecular parameters need to be optimized to better select patients. Other unsolved questions remain, such as the study of the efficacy of the drugs in a sufficient number of pediatric patients and the use of pharmacodynamic models to help to define response predicting factors for these specific targeted agents. As these treatments will enter routine practice over the next few years, overcoming the challenges associated with the rational use of targeted PI3K anticancer therapies will help evolve the next generation of personalized pediatric oncology care.

## Conclusion

In summary, our clinical experience with agents that target the PI3K pathway is growing. Lessons from adult and pediatric trials underscore the importance of incorporating agents that target specific tumorigenic pathways in rationally designed combinatorial regimens, especially when there is strong mechanistic rationale and preclinical evidence of synergistic activity. Preclinical comprehensive trials that incorporate pharmacokinetics, tumor biomarkers to assess molecular targets, and a relevant dosing schedule with metrics that mirror clinical evaluations for assessment of tumor response can help identify the targeted agents and combinations with the best potential for moving from the bench to the bedside in future clinical trials.

## Conflict of Interest Statement

The authors declare that the research was conducted in the absence of any commercial or financial relationships that could be construed as a potential conflict of interest.
